# Gender Differences in Individual Dishonesty Profiles

**DOI:** 10.3389/fpsyg.2021.728115

**Published:** 2021-12-10

**Authors:** Adrián Muñoz García, Beatriz Gil-Gómez de Liaño, David Pascual-Ezama

**Affiliations:** ^1^Department of Methodology and Social Psychology, Universidad Autónoma de Madrid, Madrid, Spain; ^2^Department of Experimental Psychology, Cognitive Processes, and Speech Therapy, Universidad Complutense de Madrid, Madrid, Spain; ^3^Center for Biomedical Technology, Universidad Politécnica de Madrid, Madrid, Spain; ^4^Accounting and Financial Administration Department, Universidad Complutense de Madrid, Madrid, Spain

**Keywords:** dishonesty, gender differences, dishonesty classification, die task, experimental

## Abstract

Dishonesty has an enormous impact on all aspects of our society. It causes huge financial losses annually, so efforts to understand dishonest behavior have increased. However, one of the main questions yet to be answered is whether dishonesty varies according to gender. Do men behave more dishonestly than women? Although the literature points to a yes, there is still no consensus on the matter. We examined gender differences in dishonesty in a large sample (*N* = 2,452) using a model recently developed by Pascual-Ezama et al. It is a variation of the classic *die-under-the-cup* task. It enabled us to identify individual dishonesty profiles and look for gender differences between them. The results show that the men were more prone to behave dishonestly than women with small rewards, who seem satisfied without maximizing the potential reward. However, the differences vanished when there was no reward. The men also showed more radical dishonest behavior than the women. The results also suggest that gender differences might be shaped by factors other than gender.

## Introduction

Whether we like it or not, dishonesty seems to be inherent in the human condition. Unfortunately, dishonest behavior is a daily occurrence at every level of life: at work, at home, at school, and in various social settings. It is so common that 93% of the 2,624 participants in an extensive poll in 2004 reported different types of daily dishonest behaviors (Kalish, 2004). However, despite the everyday nature of dishonesty and its social acceptance in certain cultures, it has an enormous impact on economies (e.g., Mazar and Ariely, 2006) such that annual losses were once estimated to have reached around $52 billion in workplaces in the United States alone (Weber et al., 2003). It also affects social policy, education, and personal wellbeing (e.g., Christensen and Wright, 2018; Lee et al., 2020). It is therefore not difficult to see why dishonesty research has grown rapidly in recent years. The complex nature of dishonesty, which is sensitive to external and internal factors in human interactions, means that we lack a comprehensive general model of dishonest behavior. Jacobsen et al. (2018) conducted a review of dishonesty research, offering an insightful guide to dishonest behavior. However, although they described some of the major advances in dishonesty research to date, they also raised critical questions that need to be addressed: Why do we cheat? What scenarios elicit dishonesty? Who is more prone to dishonesty? What factors drive dishonesty? It may be suggested that gender is one, although empirical studies are not conclusive. The present study aimed to shed some light on the matter. Are there gender differences in dishonesty? If so, how are they manifested?

Several pre-1990s studies (e.g., Eisen, 1972) showed that, in general, men seemed to show higher levels of dishonesty than women. Ward and Beck (1990) argued that this difference might have resulted from women’s propensity to follow social rules, as the sex-role socialization theory suggested. More importantly, the authors suggested that women also cheated when they were allowed to do so. Using the die-under-the-cup task (wherein participants must roll a die and, depending on the outcome reported, they can gain higher or lower rewards), Fosgaard et al. (2013) observed that the women reached the same level of dishonesty as the men when they were reminded that they could cheat. These results implied that the men were somehow more aware of the chance to lie than women. Indeed, more recent studies have found no differences between males and females in terms of dishonest behavior (e.g., Ezquerra et al., 2018; Siniver, 2021).

Others (Gino et al., 2013) showed that females cheated more than men in certain tasks. Using a math-based task, the authors argued that the women may have cheated more to compensate for the general belief that women perform worse in maths. So, despite the bulk of studies claiming that men are more likely to engage in dishonest behavior (e.g., Capraro, 2017), the findings are contradictory. The evidence thus far suggests that the factors driving gender differences have yet to be elucidated (see Rosenbaum et al., 2014 for a review).

Some factors have already been presented. For instance, as mentioned above, Gino et al. (2013) suggested that the belief that women are worse at maths tasks may have explained why they cheated more. Thus, *perceived competence* seems to be related to the proneness of their dishonest behavior. According to Maggian and Montinari (2017), high-performing competitive females are more likely to be dishonest. Competition has been discussed as a factor mediating gender differences. Schwieren and Weichselbaumer (2010) reported an increase in women’s cheating within a competitive setup compared with a non-competitive one, whereas men’s remained stable across both. However, this does not mean that men are not also influenced by competition. Nieken and Dato (2016) ran a task in which participants, paired with anonymous peers, only received rewards when they reported better outcomes. The males claimed better outcomes than the women and were thus likely to have cheated more. In that instance, the presence of a direct peer/competitor seemed to make the men cheat more than the women. Muehlheusser et al. (2015) claimed that men in groups were more likely to cheat than females in groups. Interestingly, when decisions had to be made on an individual basis, differences between the genders disappeared, as Muehlheusser et al. (2015) also discovered. Houser et al. (2016) presented evidence that parents were more honest in front of their daughters than in front of their sons. Erat and Gneezy (2012) concluded that women were more likely to tell an altruistic *white lie* (i.e., a lie that benefits the counterpart even if it entails a slight loss to oneself) but were less likely than men to engage in a Pareto white lie (i.e., a lie that benefits both parties). Finally, planning seems to be a factor involved in gender-dishonesty interactions. Chowdhury et al. (2021) argued that men lie more than women when an unexpected opportunity arises to do so.

However, a factor that has not been tested yet is based on the type of dishonest behavior elicited *per se*. Most previous studies analyzed aggregated data, but they did not determine which, and under what conditions, individual subjects were cheating or lying. We took a novel approach in our study. We looked at dishonesty at a personal level to determine the nature of the particular dishonest behavior to compare reported versus real outcomes and thus establish direct comparisons between men and women. Based on the die-under-the-cup task, which was first proposed by Fischbacher and Föllmi-Heusi (2013), and following the Pascual-Ezama et al. (2020) paradigm, we asked participants to roll a virtual die using their mobile devices (cellphones, tablets, or similar). We controlled for gender (see Dufwenberg and Gneezy, 2005 or Shalvi et al., 2011 for similar designs). The die-under-the-cup task involves participants rolling a die in private to earn a reward. The reward depends on the outcome they report; they can deceive either to earn the reward or to increase the outcome reward. To measure dishonesty individually, Pascual-Ezama et al. (2020) proposed a variation of the task that allows the researcher to discover the real distribution of the rolls. We explain the procedure in more detail in the section “Materials and Methods.” Using this new approach, Pascual-Ezama et al. (2020) presented a new classification for individual dishonesty profiles. In addition to the lucky individuals who obtained the highest reward by chance, they found other behavioral profiles for those less fortunate. There were two types of honest people: “unlucky honest,” who had no reward; and “lucky honest,” who had a reward and claimed their winnings from having rolled the die. Excluding honest and lucky people, there were three different types of dishonest participants: the “cheating-non-liars” were those who reported a real-outcome, but cheated rolling the die several times until they reached the desired reward, contrary to the rules (they could only roll the die once); the “liars,” who directly lied and claimed a reward they did not deserve when rolling the die; and the “radically dishonest,” who did not even roll the die but claimed the maximum reward. Within each of these three categories, some maximized the reward, and others did not. The study aimed to determine whether similar profiles would be found for men and women. If so, how were they distributed within them? Did any potential gender differences change according to the profile? The results showed differences between men and women only within some of them.

## Experiment

### Materials and Methods

#### Participants

To guarantee sufficient analytical power, we decided to run the experiment with a significantly sized sample of more than 2,000 participants (Fox et al., 2009; García-García et al., 2013). The 2,452 individuals (1,286 males and 1,166 females) were recruited by Amazon Mechanical Turk, and they received $1.50 for turning up and an opportunity to earn up to $0.50 performance-based bonus in the first part of the experiment. One hundred-and-twenty-six participants (76 men and 50 women) did not complete the task correctly (i.e., they did not complete the MTurk process with the MTurk code), so they were eliminated. Another 324 participants (212 men and 112 women) were excluded in accordance with Pascual-Ezama et al.’s (2020) criterion^1^. 29 participants (17 male and 12 female) report less than they obtain. We consider these participants as “incoherent”; the rest did not use the suggested website, so we could not get sufficient information from them. Respect the “incoherent” participants, it could be a mistake when they report, it is possible they do not understand the instructions correctly, or we can suppose any logical reason. Perhaps they have extreme social image concerns, and they believe that someone reporting a five is seen as “most likely dishonest.” Then they might consider reporting a 4. In that way, they only give up a small amount of money but gain a lot in the social image dimension. In any case, as we do not have information about the real reason, the number of excluded participants is minimal, and even if they had lied, they would not have done it with dishonest intentions but for self-image. Therefore, we decided to eliminate them. Concerning the rest of excluded participants, we cannot classify them as we do not have enough information. However, we have analyzed the distribution of reported outcomes to rule out a possible selection effect (see section “Results”). The final sample comprised 2,002 participants, of whom 1,004 were women and 998 were men; the average age was 34 (*SD* = 13) and 36 (*SD* = 11), respectively.

#### Procedure

The experiment was conducted using the MTurk platform, and the participants were paid after submitting their report^2^. The experiment consisted of an adaptation of the die-under-the-cup task proposed by Fischbacher and Föllmi-Heusi (2013), using the new paradigm proposed by Pascual-Ezama et al. (2020). Participants were asked to roll the die on www.rollandflip.com or a similar website using their cell phone. They can use our suggested website to roll the die^3^ or any other website to roll a die, but importantly they use their cellphone, so the perception of no-supervision is high. They would receive no bonus if they rolled a 6, following Fischbacher and Föllmi-Heusi’s (2013) rewards system. They could therefore choose not only to be (dis)honest but also to adapt their (dis)honesty according to different levels, from the maximum to minimum rewards (see Table 1). Each participant received the same simple and short instructions: “First, ensure you have a smartphone, a tablet, or another electronic device with internet access. You have to roll a die, and you can earn money depending on your roll result: if you roll a 1, you will receive $0.10. If you roll a 2, you will receive $0.20. If you roll a 3, you will receive $0.30. If you roll a 4, you will receive $0.40. If you roll a 5, you will receive $0.50. If you roll a 6, you will receive nothing. Now, please proceed to the following website: https://www.rollandflip.com/ (or another similar site), select the “roll the die” option, and roll the die once.” The critical manipulation here was to link the real outcome and the reported one for a given person. We had access to the rollandflip.com database to match the rolls individually, controlling the exact moment every participant performed the task. Although we could consider that deception occurs place since participants maintain the perception of impunity while the researchers are monitoring their behavior, this procedure used by Pascual-Ezama et al. (2020) is essential to classify the different behavioral profiles. Therefore, we could determine the precise number of rolls and the real outcome distribution and link them with the reported ones for each participant. Most of the participants chose to use the rollandflip.com website, allowing us to connect their real and reported outcomes to study honest and dishonest behavior in detail. The website www.rollandflip.com was created by researchers to record real outcomes from rolling a die or flipping a coin. We were able to record the real results, IP address, timestamp, the reported results, and the time the participants took to complete the task. Therefore, we were able to link data from https://rollandflip.com with https://behavioralexperiments.com to classify participants’ real behavior.

**TABLE 1:** Dishonesty classification.

Behavior	Label	Classification
Roll the die − obtain 5 − report 5	LUCKY	Lucky

Roll the die − obtain 1 to 4 − report the same outcome obtained		Lucky honest
	HONEST	
Roll the die − obtain 6 − report 6		Unlucky honest

Roll the die − obtain 6 − roll several times until other outcome and report it		Sub-maximizing cheaters non-liars
	CHEATERS	
Roll the die – obtain an outcome different than 5 − repeat until 5 − report 5	NON-LIARS	Maximizing cheaters non-liars

Roll the die − obtain an outcome − report a higher outcome, but less than 5		Sub-maximizing liars
	LIARS	
Roll the die − obtain an outcome different than 5 − report 5		Maximizing liars

Do not roll the die at all − report < 5		Sub-maximizing radically dishonest
	RADICALS	
Do not roll the die at all − report 5		Maximizing radically dishonest

*Adapted from Pascual-Ezama et al. (2020).*

## Results

The most relevant results are presented in the following three subsections. First, we show the typical population-level analysis as aggregated data from the reported results as if we did not have the real outcomes to make direct comparisons with previous studies in the field. Then, we show the individual-level analyses comparing reported and real outcomes. Finally, we group the subcategories of the (dis)honest classification into higher categories (by the dichotomy dishonest/honest, nature, and gradient); in each case, the men are compared with the women.

### Population-Level Analysis

**FIGURE 1:**
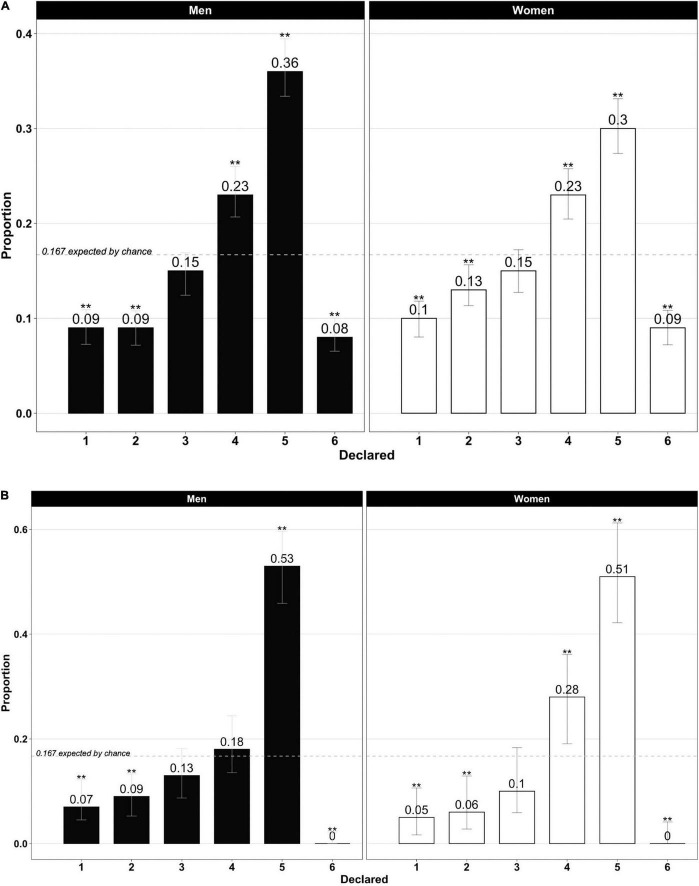
**(A)** Declared die outcome (men vs. women). Proportion test confidence interval at 95% is represented in gray. Asterisks above the confidence interval mean significant differences between the observed and expected distribution by chance. **p < 0.01; *p < 0.05. **(B)** Declared die outcome (men vs. women in excluded participants). Proportion test confidence interval at 95% is represented in gray. Asterisks above the confidence interval mean significant differences between the observed and expected distribution by chance. **p < 0.01; *p < 0.05.

We examined whether the reported outcome distribution for the males and females differed from a uniform distribution, as is the case in classical inferred tasks aggregated analyses. A Kolmogorov–Smirnov (KS) test for one sample showed that both sample distributions differed significantly from the expected uniform distribution (*p* < 0.001), which indicated that both the men and women did not report the real outcome at the first die-roll; that is, they lied or cheated. Then, we tested for each die outcome to see whether the proportions differed from what would be expected by chance. As we can see in Figure 1, high reward proportions were significantly higher than expected by chance (“4” and “5” outcomes; i.e., above the expected 16.7% by chance, as shown in the dashed lines). Low reward proportions were significantly lower than the 16.7% percentage expected by chance (“1” and “2” outcomes). Also, “6-no reward” was significantly lower than expected by chance for both the men and women. The “in-between 3” outcome fell somewhere between 14 and 15% for the men and women; however, in this case, the proportions were not significantly lower than the chance level. The KS test showed only marginally differences between the men and women (*p* = 0.08), which indicated that they cheated almost similarly. The main difference appears when analyzing results for maximizing “5” outcomes, that is, the maximum reward, although both are significantly above the chance level, men maximized the reward more than women (36% vs. 30% in outcome 5; χ^2^ = 8.37, *p* < 0.01; see Figure 1 again). There were also differences for those declaring “2”: the women reported significantly more “2s” (χ^2^ = 9.96, *p* < 0.01).

Concerning the participants eliminated for not using the proposed website and, therefore, not having information to classify them as honest or dishonest, they maintain a similar distribution (see Figure 1) to the rest of the participants, thus ruling out a sample selection effect in terms of distribution. Perhaps we could highlight that they could be a more dishonest sample. They have a higher (and unusually high) number of the maximum prize, any of them report the non-reward output (impossible from a statistical point of view), and one-third of the participants spend less than 30 s, a short time to search for a website to roll the die or to search for a physical die and respond to the experiment. In any case, we do not have enough information to classify them, so eliminating them is the most correct and conservative.

### Individual-Level Analysis

The population-level analysis revealed dishonest behavior but did not discriminate between different dishonest profiles. Although we can infer more maximizing cheating among the men than the women (the results from outcome “5”), the aggregated results did not show any gender differences when the general cheating behaviors were compared. Individual-level analyses made it possible to provide a more fine-grained picture of different forms of dishonesty, and this helped us to detect the potential gender differences that had been glimpsed in the population-level results.

First, when we surveyed the participants who were eliminated because they did not follow the rules, we observed a greater number of men; of the 324 excluded participants, 212 were men and 112 were women; χ^2^ = 19.442, *p* < 0.01). In reality, they were eliminated conservatively. Beyond having exceeded a certain time and not using the recommended website, we did not know how they behaved. However, there was a high probability that a large proportion were radicals who took longer than the time limit we considered appropriate to classify them as such. This result was therefore logical and supported the finding that the men were more radical than the women.

**FIGURE 2:**
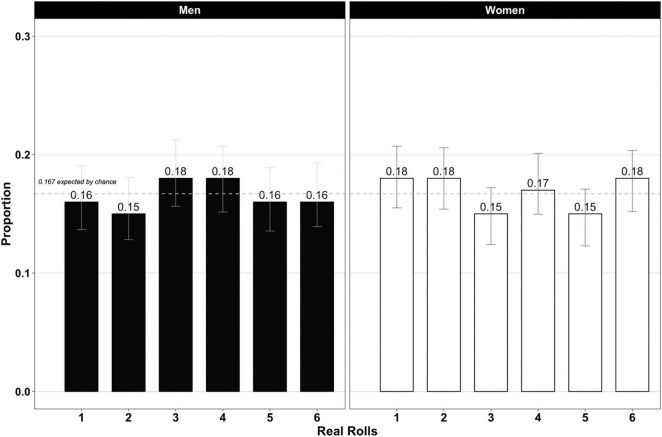
Real die outcome (men vs. women). Proportion test confidence interval at 95% is represented in gray.

Second, the real distribution did not differ from the uniform expected distribution, and nor did the proportions (see Figure 2). This is important because it shows that the theoretical distribution existed in reality, so we could take the previous deviant declared distribution as proof of a pattern of general dishonesty (see Supplementary Figure 1). We did not find any difference between genders in the proportion of real roll. Therefore, statistically speaking, the men and women started from the same conditions.

Once we had checked the statistical assumptions that allowed us to compare men and women, we linked each participant’s real outcomes with the reported outcomes following Pascual-Ezama et al. (2020) paradigm. There were no differences in the rate of “lucky” participants by gender (χ^2^ = 0.05, *p* = 0.83). There were also no differences between the men and women when there was no reward (i.e., they achieve an outcome of “6.” We then calculated the percentage of men and women reporting a different outcome than which they obtained in the first roll of the die (thus, the rate of dishonest individuals divided according to gender). The results showed that 52.2% of the men and 41.0% of the women were dishonest. The statistical analysis of relative risk (RR) revealed that the men were more dishonest than the women; in particular, the men were 1.25 times more likely to be dishonest [RR = 1.24, 95% CI (1.13, 1.35), χ^2^ = 22.6, *p* < 0.001].

**FIGURE 3:**
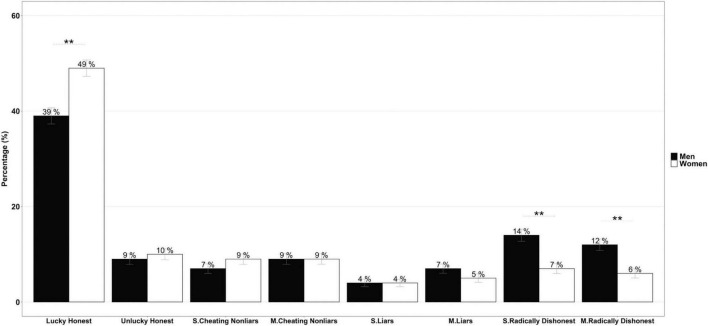
Percentage of men (black) and women (white) for each (dis)honesty profile. Note that lucky people achieving an outcome “5” were excluded from the analysis. In contrast, honest people included here are those achieving an outcome other than “5”; S., sub-maximizing and M., maximizing. The number above bars indicates the percentage; asterisks indicate significance in pairwise proportion comparisons: **p < 0.01.

We calculated the percentages again, but for each profile, as described in Pascual-Ezama et al. (2020), to obtain a more detailed and at the same time broader picture of the nature of the dishonest behavior of the men and women. In Table 1, we describe each of those profiles, and in Figure 3, we can see the percentage of participants at each profile divided by gender (besides the “lucky,” who, as we explained above, were removed from the individual analysis since they did not provide sufficient information for the study).

**FIGURE 4:**
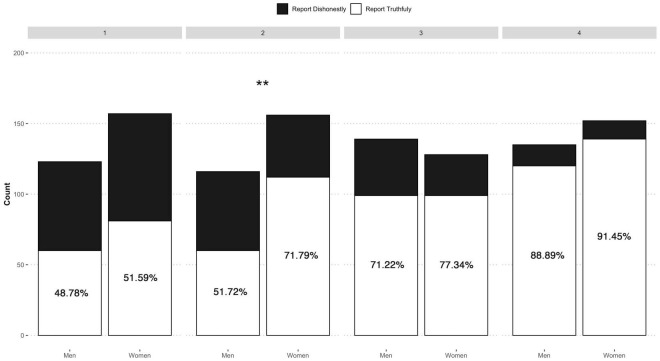
Lucky-honest declared die outcome (men vs. women). Asterisks indicate significance in pairwise proportion comparisons: **p < 0.01.

As Figure 2 shows, there were two significant results. First, the percentage of “radically dishonest” (i.e., those who did not even roll the die) was higher for the men, both for non-maximizers [RR = 1.83, 95% CI (1.37, 2.44), χ^2^ = 17.12, *p* < 0.001] and maximizers [RR = 1.83, 95% CI (1.34, 2.49), χ^2^ = 14.53, *p* < 0.001]. That is, regardless of maximizing or not, the men were more “radically dishonest” (see Figure 2). Second, for the honest people, the differences between the men and women were only apparent among the “lucky honest.” There was a significantly higher proportion of “lucky honest” women than men [RR = 1.27, 95% CI (1.14, 1.42), χ^2^ = 19.42, *p* < 0.001]. These results suggest that when obtaining a “minimum” reward (“1−4”), the women seemed to be sufficiently satisfied to behave honestly. We hypothesized that the men needed a higher reward to act honestly. To test this, we analyzed the frequency of outcomes (1, 2, 3, or 4) for the “lucky honest” people according to gender (Figure 4). Although the outcome “4” was the most often declared among both groups, the same occurs for the rest of outcomes. Regardless of the number of times the participants (men and women) got each of the outcomes, women honestly reported a higher percentage. The trend is unanimous for the different outcomes and significant for outcome 2 (χ^2^ = 11.53, *p* < 0.01). This result could be because as there are more male radicals, more women are throwing the die, and therefore, we could find a more significant number of lucky honest women. However, in this case, we should also find a higher number of women in the other groups, and this is not the case. It could also be the case that the proportion of female rolls is higher in the outcomes with prizes, and therefore more women will accept smaller outcomes. However, as we can see in Figure 2, this has not occurred either. Therefore, the results supported our hypothesis that the women were probably more satisfied with smaller rewards.

**FIGURE 5:**
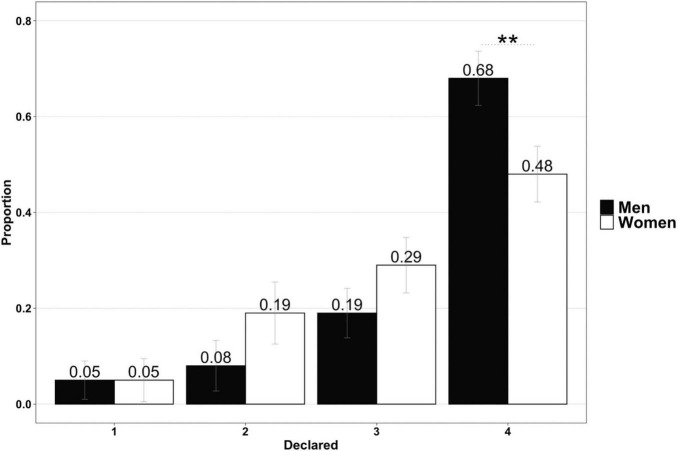
Sub-maximizing cheating non-liars die outcomes obtained after several rolls (men vs. women). Error bars are represented in gray. Asterisks indicate significance in pairwise proportion comparisons: **p < 0.01.

If this was indeed the case, there should also have been differences between the men and women in the sub-maximizing cheater category. In particular, there should have been a greater proportion of women cheating with outcomes “2” or “3,” with men tending to wait for “4” outcomes to cheat more frequently. As Figure 5 shows, this was the case: there are significantly more cheating men waiting for a “4” outcome (0.68), compared with women (0.48). It should be remembered that in this case, the participants cheated by rolling the die several times until they obtained the desired outcome/reward (χ^2^ = 5.37, *p* = 0.02). Although the differences in outcome “3” were not significant (χ^2^ = 1.31, *p* = 0.25), the tendency was again more apparent among the men, who were more inclined to be satisfied with a “4” outcome. The women were more frequently satisfied with lower outcomes.

### Category-Level Analysis

We merged the dis(honest) labels into three categories, again based on Pascual-Ezama et al. (2020; see Table 1). First, as defined in Figure 3, the honest people comprised those considered lucky and unlucky. Second, we took into account the nature of the dishonest behavior regardless of the gradient of dishonesty (i.e., whether the behavior was maximized or not). Second, we combine cheater non-liars (i.e., those who rolled the die several times until they obtained the desired value); liars (i.e., those who reported a different outcome to the one obtained from rolling the die); and radicals (i.e., those who did not even roll the die and reported the desired outcome to win the reward). The third group comprised, according to the gradient of dishonesty, those who maximized their dishonest behavior (reporting the outcome “5”), namely, the *maximizers*; and those who decided to report a different outcome from “1” to “4” (which probably fitted the minimum outcome they considered before claiming a reward), namely, the *sub-maximizers*. In Table 2, we can see the proportion of women and men who occupied each of those merged profiles, as well as the chi-square tests that illustrated the significant differences between them.

**TABLE 2:** Proportion of men and women with different (dis)honesty profiles.

		Women	Men	Chi-squared test
Honest		59%	48%	$\chi_{1,N=1753}^{2}=22.69,p<0.0001$
Dishonest		41%	52%	
By gradient	Sub-maximizers	50%	47%	$\chi_{1,N=814}^{2}=0.55,p=0.46$
	Maximizers	50%	53%	
	Cheaters	44%	31%	$\chi_{2,N=814}^{2}=19.22,p<0.0001$
By nature	Liars	22%	20%	
	Radicals	34%	49%	
Total (*n*)		877	876	

*Chi-squared test reports independence between honest and dishonest participants; sub-maximizers and maximizers; and liars, cheaters, and radicals with a 95% confidence level.*

Analyzing differences by profile, we can see that, first, the difference between men and women in terms of the percentage of individuals exhibiting honest or dishonest behavior was significant: the women were more honest. Second, depending on the nature of the dishonest behavior, there were again differences between the men and women (see the third chi-square test in Table 2). Still, this only applied to the radicals: the men were 1.83 times more radical than the women [RR = 1.83, 95% CI (1.50, 2.23), χ^2^ = 36.2, *p* < 0.001]. No gender differences were apparent in the proportion of cheaters non-liars or liars. Finally, there were no differences between men and women regarding the gradient of dishonesty, whether the rewards were maximized or not. While these results were not significant, there were differences in outcomes in the earlier analysis.

## Conclusion and Discussion

Although various studies have pointed toward a difference in dishonest behavior between men and women (often showing men as more prone to behave dishonestly), the nature of this difference has not been studied in detail. Certain factors, such as “perceived competence,” “individual” versus “grouped” dishonesty, competition, or planning dishonest behavior have revealed a more diverse picture showing—in some cases—no gender disparities and even situations in which women are more dishonest than men. The present study aimed to explore those potential gender differences in dishonesty in more detail by using the Pascual-Ezama et al. (2020) paradigm and ensuring that the participants did not know they were being observed (see Fries et al., 2021). Under this paradigm, we were able to study the nature of different types of dishonest behavior (e.g., cheating, lying, and radically dishonest actions) and its gradient (i.e., maximizing or otherwise). We were also able to depict dishonesty at an aggregated level, as previous studies have done, but more importantly, at an individual level. We examined how the participants behaved by collecting and comparing self- and real reports using the die-under-the-cup online task. The results showed that women were more honest than men in general, but depending on the nature of the dishonest behavior, they could behave similarly or in distinctive ways by graduating their actions.

In particular, we observed that the women were more likely to be honest for lower rewards, while the men needed higher rewards to maintain honest behavior. The women seemed to be satisfied enough at lower rewards, which led them to decide not to cheat for higher ones (see Figures 2, 3). The men tend to maximize rewards, even at non-maximizing levels (that is, achieving outcomes “3” and “4”). Even when cheating, the women tended to be satisfied with lower rewards than the men, indicating that they seemed to be more satisfied even when they were actually cheating (see Figure 4). Other studies found that men over-reported higher results than women (e.g., Nieken and Dato, 2016; Grosch and Rau, 2017; Abeler et al., 2019; Benistant et al., 2021). Our results revealed that there were more radically dishonest men than women [according to the Pascual-Ezama et al. (2020) classification], which again supported the idea that the women were more satisfied with lower rewards.

What is also significant is that we replicated Pascual-Ezama et al.’s (2020) dishonesty profiles using a sample of around 2,500 participants. Moreover, we replicated the traditional general finding that men are more dishonest than women, even at the aggregate level. By using Pascual-Ezama et al.’s (2020) new model, we could go beyond individual levels of analysis to observe both real and reported outcomes. Under these circumstances, although differences between men and women were apparent, there were no differences when there were no rewards: The proportion of the unlucky honest was statistically the same for both the men and women. In other words, the men’s levels of honesty did not differ from the women’s when they were not winning anything. This result accords with the literature. Our results add a significant nuance to the standard interpretations of differences between men and women regarding dishonesty. Our null results and previous results suggest that gender differences are reliant on the reward factor; differences in rewards reveal differences in gender dishonesty. Although more research is needed, our rewards were small enough to generate results that were different than when higher rewards were available and higher differences obtained between the different outcomes in the die-under-the-cup task. It seems that rewards can modulate gender differences in dishonesty, in that men may be prepared to be more radical in their quest for higher rewards, and women are more satisfied with lower rewards.

## Data Availability Statement

The raw data supporting the conclusions of this article will be made available by the authors, without undue reservation.

## Ethics Statement

The studies involving human participants were reviewed and approved by the Comité de Ética de la Investigación UAM. The patients/participants provided their written informed consent to participate in this study.

## Author Contributions

AM and DP-E developed the study concept and performed the testing and data collection. AM drafted the manuscript. BG-G provided critical revisions. All authors contributed to the data analysis, interpretation, and study design, and approved the final version of the manuscript for submission.

## Conflict of Interest

The authors declare that the research was conducted in the absence of any commercial or financial relationships that could be construed as a potential conflict of interest.

## Publisher’s Note

All claims expressed in this article are solely those of the authors and do not necessarily represent those of their affiliated organizations, or those of the publisher, the editors and the reviewers. Any product that may be evaluated in this article, or claim that may be made by its manufacturer, is not guaranteed or endorsed by the publisher.

## Footnotes

1 Individuals who gave an immediate response (less than 5 s after receiving computerized instructions) without using www.rollandflip.com (see the following section) were classified as radically dishonest because they claimed the high money outcome without flipping a coin or rolling a die on some other website (5 s was not enough time to go to an alternative website and/or initiate a coin toss or a die roll). Individuals who submitted a report that took more than 5 s and also did not go to our chosen website were eliminated from the final sample.

2
https://behavioralexperiments.com

3
www.rollandflip.com

## Supplementary Material

Supplementary Figure 1 Real and declared rolls. Real rolls in the horizontal axis and declared roll in the vertical axis.

Supplementary Figure 2 Average report for different profiles (Maximizers report always the maximum outcome “5”).
