# Supplementary figures and images for: Gender Differences in Individual Dishonesty Profiles
Source: Front Psychol. 2021 Dec 10;12:728115. doi: 10.3389/fpsyg.2021.728115 (PMC8703141; doi:10.3389/fpsyg.2021.728115)

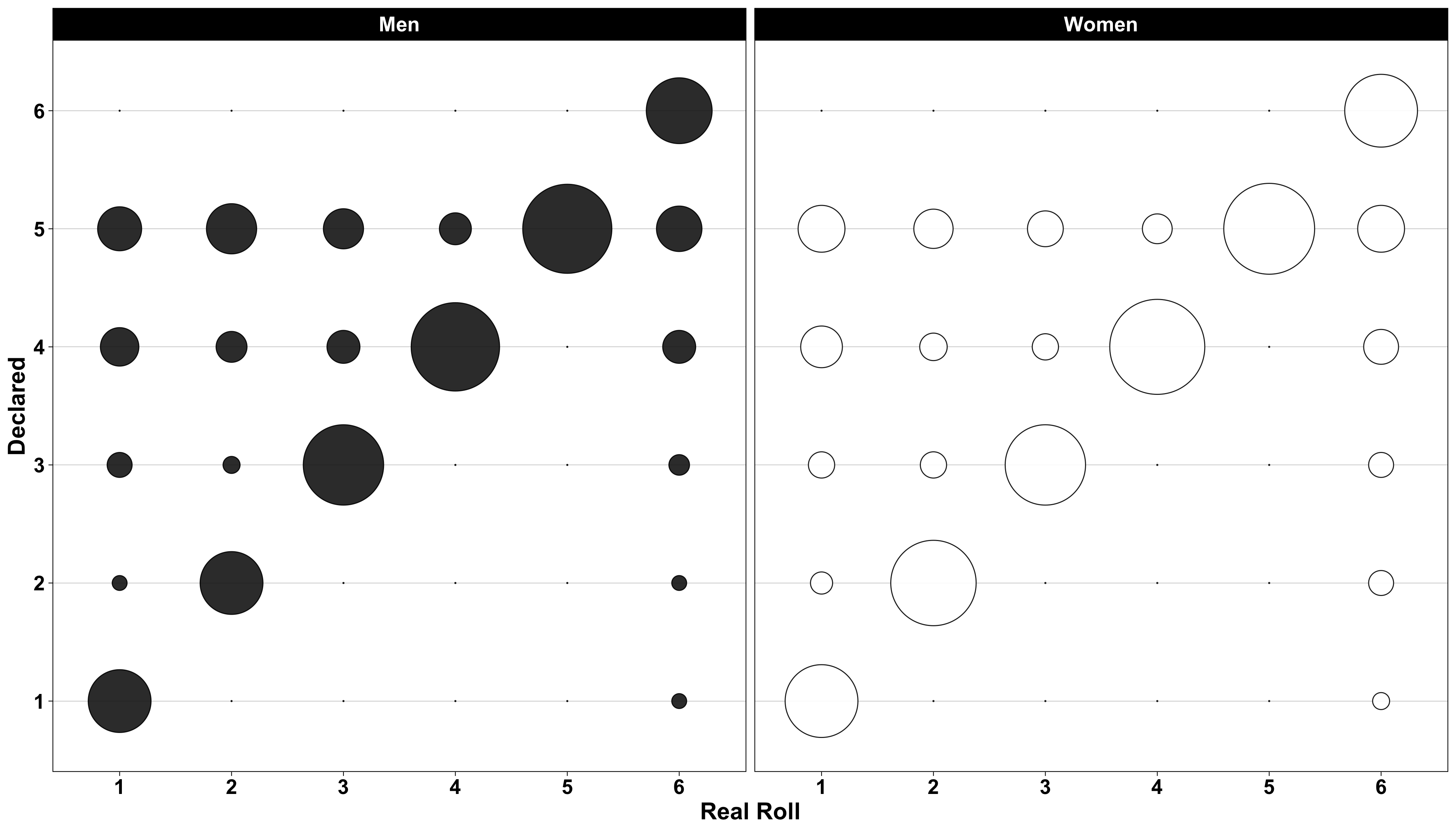

Supplement: Supplementary Figure 1 — Real and declared rolls. Real rolls in the horizontal axis and declared roll in the vertical axis. [file Image_1.png]

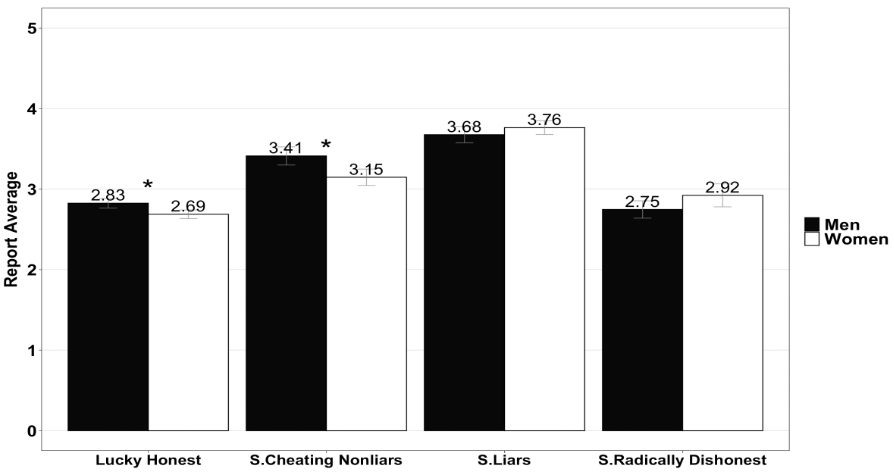

Supplement: Supplementary Figure 2 — Average report for different profiles (Maximizers report always the maximum outcome “5”). [file Image_2.png]
